# Adherence to ready-to-use food and acceptability of outpatient nutritional therapy in HIV-infected undernourished Senegalese adolescents: research-based recommendations for routine care

**DOI:** 10.1186/s12889-020-08798-z

**Published:** 2020-05-15

**Authors:** Fatou Niasse, Marie Varloteaux, Karim Diop, Sidy Mokhtar Ndiaye, François Niokhor Diouf, Pape Birane Mbodj, Babacar Niang, Aminata Diack, Cecile Cames

**Affiliations:** 1 Comité national de lutte contre le sida (CNLS), Dakar, Sénégal; 2 Institut de recherche pour le développement (IRD), UMI233 TransVIHmi, INSERM U1175, Université de Montpellier, 911 Av Agropolis, 34394 Montpellier, France; 3 Centre régional de recherche et de formation à la prise en charge clinique (CRCF), Dakar, Sénégal; 4 Division de lutte contre le sida et les infections sexuellement transmissibles (DLSI), Ministère de la santé et de l’action sociale, Dakar, Sénégal; 5 Centre hospitalier régional, Ziguinchor, Sénégal; 6 Centre hospitalier régional, Kaolack, Sénégal; 7 Centre Hospitalier National Universitaire d’Enfants Albert Royer, Dakar, Sénégal

**Keywords:** Acute malnutrition, Acceptability, Adherence, HIV, Ready-to-use food, Children, Adolescents, Africa

## Abstract

**Background:**

Ready-to-use food (RUF) is increasingly used for nutritional therapy in HIV-infected individuals. However, practical guidance advising nutrition care to HIV-infected adolescents is lacking, so that little is known about the acceptability of such therapy in this vulnerable population. This study assesses the overall acceptability and perception of a RUF-based therapy and risk factors associated with sub-optimal RUF intake in HIV-infected undernourished adolescents in Senegal.

**Methods:**

Participants 5 to 18 years of age with acute malnutrition were enrolled in 12 HIV clinics in Senegal. Participants were provided with imported RUF, according to WHO prescription weight- and age-bands (2009), until recovery or for a maximum of 9–12 months. Malnutrition and recovery were defined according to WHO growth standards. Adherence was assessed fortnightly by self-reported RUF intake over the period. Sub-optimal RUF intake was defined as when consumption of the RUF provision was < 50%. RUF therapy acceptability and perceptions were assessed using a structured questionnaire at week 2 and focus group discussions (FGDs) at the end of the study. Factors associated with sub-optimal RUF intake at week 2 were identified using a stepwise logistic regression model.

**Results:**

We enrolled 173 participants, with a median age of 12.5 years (Interquartile range: 9.5–14.9), of whom 61% recovered from malnutrition within the study period. Median follow-up duration was 66 days (21–224). RUF consumption was stable, varying between 64 and 57% of the RUF provided, throughout the follow-up. At week 2, sub-optimal RUF intake was observed in 31% of participants. Dislike of the taste of RUF (aOR = 5.0, 95% CI: 2.0–12.3), HIV non-disclosure (5.1, 1.9–13.9) and food insecurity (2.8, 1.1–7.2) were the major risk factors associated with sub-optimal RUF intake at week 2. FGDs showed that the need to hide from others to avoid sharing and undesirable effects were other constraints on RUF feeding.

**Conclusions:**

This study revealed several factors reducing the acceptability and adherence to RUF therapy based on WHO guidelines in HIV-infected adolescents. Tailoring prescription guidance and empowering young patients in their care are crucial levers for improving the acceptability of RUF-based therapy in routine care.

**Trial registration:**

ClinicalTrials.gov identifier: NCT03101852, 04/04/2017.

## Introduction

Malnutrition is common among children living in low incomes settings and is even more critical in HIV-infected children, including those on antiretroviral treatment (ART) [1, 2], as HIV infection can induce growth failure and malnutrition in children, which in turn contribute to faster disease progression. Successful treatment of acute malnutrition in young children < 5 years [3], including HIV-infected children [4–6], has been achieved using Plumpy Nut® and Plumpy Sup® (Nutriset, Malaunay, France) food supplements, developed for pediatric use and generically referred to as imported ready-to-use food (RUF). During the last decade, most African countries adopted national guidelines for RUF-based treatment of acute malnutrition in children < 5 years, including those with HIV.

The vulnerabilities of HIV-infected adolescents, especially those with perinatal HIV infection, have been extensively described [7]. They often have more advanced HIV disease with related co-morbidities associated with delayed HIV treatment and lifelong infection [8]. Perinatally HIV-infected children ≥5 years and adolescents are also vulnerable to malnutrition and often face severe forms. Recent studies reported 16 to 33% of acute malnutrition in children and adolescents 5 to 19 years-old on long term ART, of whom one-third was severely undernourished [9, 10]. Because National health systems have been oriented towards monitoring and care of children < 5 years and adult, adolescents health care services, including those living with HIV, suffer from multiple deficiencies, including the lack of nutritional care [11]. Despite World Health Organisation (WHO) recommendations for the integration of nutritional interventions into comprehensive HIV treatment programmes [12], nutritional therapy at HIV treatment sites in sub-Saharan Africa remains poorly available [13]. This is particularly true with respect to the recommendations for adolescents, 2009 WHO Preliminary guidelines for an integrated approach to the nutritional care of HIV-infected adolescents [14].

Consequently, acceptability data and practical guidance advising the implementation of nutrition care in these vulnerable patients are lacking. This is of concern, as the effectiveness of medical therapy is strongly associated with its level of acceptance, adherence to prescription, timing of intake, and medical and psychosocial surroundings [15], all of which may differ among patients of different age classes. Experimental data on the acceptability of RUF were obtained primarily from studies conducted in adults since RUF provisioning has become a widely used outpatient strategy among vulnerable adults through food-by-prescription programmes. Qualitative studies conducted in Southern Africa reported poor overall acceptability of RUF [16–18], whereas its organoleptic qualities were rated as good in studies from Vietnam [19] and Haiti [20]. Assessment of a pilot nutrition support providing RUF to 43 HIV-infected children and adolescents with acute malnutrition in two HIV clinics in Senegal shown that beneficiaries consumed 50% of their RUF on average and 20% defaulted from the programme [21]. Following this pilot study, the SNACS Study was implemented to assess the acceptability, effectiveness, and feasibility of outpatient RUF-based nutritional therapy among HIV-infected children and adolescents presenting with acute malnutrition across Senegal. The intervention protocol followed the 2009 WHO Guidelines [14]. The operational objective of this research, conducted in close partnership with the Senegalese Ministry of Health, was to provide the national HIV programme with evidence-based recommendations for outpatient nutritional therapy in HIV-infected children and adolescents.

The objectives of the present report are first to describe the overall acceptability of the nutritional intervention including perceptions, behaviours, and constraints surrounding RUF use. Secondly, we assess adherence to RUF prescription and investigate risk factors associated with sub-optimal intake. To our knowledge, the present study is the first to conduct such assessments among HIV-infected undernourished adolescents.

## Methods

### Study setting

The SNACS study was carried out in Senegal from April 2015 to January 2017 in 12 public paediatric clinics: two in the Dakar district, the capital city, (Albert Royer National Paediatric Hospital and Roi Baudouin Hospital) and 10 in decentralized settings (Regional Hospitals of Saint Louis, Louga, Mbour, Kaoloack, Ziguinchor and Kolda; Health centres of Thiès, Nioro du Rip, Bignona and Kolda). Study implementation was gradual, starting with the central sites in April 2015 and ending with the Kolda sites in March 2016. SNACS, which refers to “snacks”, is the French acronym for nutritional support of growing adolescents in Senegal.

### Study population and procedures

The analyses include participants aged 5 to 18 years under active follow-up for their HIV infection and presenting with acute malnutrition. Children with acute malnutrition have low weight for their height due to a rapid weight loss, while those with chronic malnutrition have a low height for their age. Moderate acute malnutrition (MAM) was defined as body mass index-for-age z-score (BMIZ) of < − 2 and ≥ − 3, and severe acute malnutrition (SAM) as BMIZ < − 3, according to the WHO growth standards [22]. Participants also were without medical complications and succeeded at an appetite test, which assesses their ability to consume a weight-appropriate portion of RUF [23].

Participants were seen every 2 weeks for clinical assessment, provision of RUF for the next 2 weeks, and monitoring of adherence to RUF over the preceding 2 weeks and provided with RUF until they recovered from acute malnutrition (BMIZ ≥ − 2), discontinued the study (default, death), and at the latest, until the study was concluded, i.e. 9 months in decentralized clinics or 12 months in Dakar. A social health worker counselled participants and caregivers to distribute RUF intake throughout the day, to eat the RUF in small bites to cope with nausea and lack of appetite, and to have sufficient drinking water at their disposal to prevent diarrhoea. Participants were also advised to avoid RUF intake during mealtimes, in order to preserve family meals and avoid sharing with other family members.

The study used imported RUF, Plumpy Nut® and Plumpy Sup®. Both are packaged in a 92 g individual sachet providing 500 kcal in the form of an energy-dense lipid paste made from peanut butter, oil, sugar and a high vitamin and mineral supplement (the first contains milk powder, while the second contains soy proteins) [24]. Plumpy Sup® is recommended in the management of MAM, amongst other options, while Plumpy Nut® is dedicated to the treatment of SAM. WHO recommends a Plumpy Nut® prescription of 75 to 100 kcal/kg/d in children aged 5 to 10 years and 60 to 90 kcal/kg/d above that age [14]. Across all ages, Plumpy Sup® was prescribed at the lowest recommended dose of 60 kcal/kg/d to participants with MAM. Prescription by weight may involve a high daily dose in older HIV-infected children and adolescents. The lowest value was used and maximum energy intake provided by RUF was limited to 2000 kcal/d i.e. 4 sachets in all participants to preserve habitual diet and prevent appetite saturation.

### Data collection and analysis

#### Participants’ characteristics at enrolment

Clinical and therapeutic characteristics of the participant were recorded from medical files while socioeconomic and food security data were collected with the caregiver. The household food insecurity access scale (HFIAS)—a list of nine specific questions about the availability and accessibility of foods and food-related worries for the household during the previous month—was used to assess food insecurity [25]. This access scale generates a 4-class variable of food insecurity—none, mild, moderate, and severe. In this analysis, we tested both the 4-class and the derived binary variable (food insecurity: yes vs. none). Minimum dietary diversity was defined as having consumed at least 5 food groups out of 10 the day before the study visit, using the Global Dietary Diversity Indicator for Women, as measured by participant 24-h recall of food consumption at enrolment and week 2 [26]. The recall was primarily conducted with the participant (with the help of the caregiver if need be).

At week 2, a structured questionnaire covering 5 topics, organoleptic appreciation of RUF, mode of intake, 24-h recall of RUF intake, self-stigma associated to RUF intake and RUF sharing, was administered primarily to participants ≥7 years or caregivers below that age. Questions about RUF sharing were administered separately to participants ≥7 years and caregivers.

#### Adherence to RUF prescription

Based on participant and caregiver reports on RUF consumption, adherence was calculated as the percentage of reported RUF sachets consumed out of the number of sachets provided at each follow-up visit. Early sub-optimal RUF intake was defined when this ratio was < 50% at week 2.

#### Risk factors of early sub-optimal RUF intake

Associations between early sub-optimal RUF intake with socioeconomic and demographic profiles, RUF acceptability data, as well as ART drug history and severity of malnutrition, were investigated using univariable regression, after controlling for collinearity, as well as stepwise logistic regression models. Explanatory variables with *P* < 0.20 from the univariable analysis were included in the multivariable models and excluded by the stepwise procedure at *P* > 0.10. The final set of variables retained were tested for interactions. Differences were considered statistically significant at *P* < 0.05. Medians are presented with their interquartile range (IQR). All statistical analyses were performed in SAS, version 9.3 (Cary, North Carolina, USA).

#### Expectations, perceptions and experiences of participants in the nutritional intervention

Focus group discussions (FGD) were conducted at the Dakar study clinics, after the end of the total study period (12 months), with a convenience subsample of participants aged at least 7 years, which aimed to be fairly representative of the study population with regards to age, sex and severity of malnutrition. Sessions were conducted in *Wolof*, the national language of Senegal, or French, by a social health worker and the doctoral student (MV) and focused on (i) participant expectations from the study, (ii) perceptions of RUF and (iii) experiences of the nutritional intervention, depending on their outcome in the study (recovery, failure, default). FGD were audiotaped and transcribed verbatim into French. The thematic analysis which consisted in coding, synthesizing and grouping sections of text from the FGDs that covered similar issues or experiences, was performed by the doctoral student (MV) in Dedoose, version 7.0.21 (Los Angeles, California, USA).

### Ethical approval

Ethics clearance for the SNACS study protocol was given by the Ethics and Regulatory Committee and the Ministry of Health in Senegal. All parents or surrogate caregivers provided written informed consent. Participants aged ≥7 years received extended information about the research and provided verbal assent [27].

## Results

### Study population

From April 2015 to September 2016, the SNACS study enrolled 184 HIV-infected participants of whom 173 were between the ages of 5 and 18 years old (median age: 12.5 years, IQR: 9.5–14.9), 104 presenting with MAM and 69 with SAM, were included in these analyses (Table 1). All but one participant had been infected through mother-to-child transmission. The mother or father was the primary caregiver in 44 and 8% of participants, respectively, while 48% lived with a surrogate caregiver who was an ascendant (grandparents, uncles, aunts: *n* = 24), collateral (siblings, cousins: *n* = 57), or a neighbour (*n* = 2). Eighty-seven per cent were on ART at enrolment for a median duration of 48 months (15–75). Only 28% had had their HIV serologic status disclosed for whom the median time since the disclosure was 11 months (5–26). HIV status disclosure was associated with virologic suppression (< 50 copies/ml, *P* < 0.0001). Sixty-one per cent of participants recovered from wasting after a median follow-up duration of 29 days (16–65), 31% failed to reach BMIZ ≥-2 during the time of the study, 6% defaulted and 2% died. The overall median follow-up duration was 66 days (21–224), which increased from 30 days (16–115) among participants presenting with MAM to 156 days (85–275) in SAM participants (*P* < 0.0001).

**Table 1:** Characteristics of HIV-infected participants and their caregivers at enrolment in the SNACS study, Senegal ^a-b^

Characteristics	< 12 years *N* = 84		≥ 12 years *N* = 89		All *N* = 173		*P* value
**Girl**	34	(40)	35	(39)	69	(40)	0.88
**Decentralized study site**	51	(61)	42	(47)	93	(54)	0.07
**Acute malnutrition**							0.04
**Moderate**	57	(68)	47	(53)	104	(46)	
**Severe**	27	(32)	42	(47)	69	(40)	
**HAZ, median IQR**	-1.7	(-2.4 – -0.8)	-2.0	(- 2.7 – - 1.2)	-1.8	(-2.6 – - 0.9)	0.09
**At school**	58	(69)	74	(83)	132	(76)	0.03
**Family status**							0.24
**Parents alive**	31	(37)	21	(24)	52	(30)	
**Mother orphan**	20	(24)	23	(26)	43	(25)	
**Father orphan**	16	(19)	25	(28)	41	(24)	
**Double orphan**	17	(20)	20	(22)	37	(21)	
**HIV disclosed**	5	(6)	44	(49)	49	(28)	< 0.0001
**ART**^**c**^							0.02
**No ART**	17	(20)	6	(7)	23	(13)	
**ART – virologic suppression**^**d**^	26	(31)	39	(44)	65	(38)	
**ART – no virologic suppression**	40	(48)	43	(49)	83	(49)	
**CD4 cell/mm**^**3**^**, median IQR**	603	(343–861)	414	(209–707)	522	(229–780)	0.04
**Caregiver school level**							0.28
**None**	36	(43)	49	(55)	85	(49)	
**Primary**	25	(30)	21	(24)	46	(27)	
**Secondary and more**	23	(27)	19	(21)	42	(24)	
**Caregiver income**							0.50
**None**	40	(48)	36	(40)	76	(44)	
**Regular**	9	(11)	14	(16)	23	(13)	
**Irregular**	35	(42)	39	(44)	74	(43)	
**Food insecure household**	65	(77)	68	(76)	133	(77)	0.88

^a^Data are N, % unless otherwise indicated

^b^Abbreviations: *HAZ* height-for-age z-score, *IQR* interquartile range, *ART* antiretroviral treatment

^c^2 missing values

^d^Virologic suppression is defined as viral load ≤50 copies/ml

### Acceptability of RUF and feeding practices at week 2

Overall, 87, 79, 85, and 80% of participants initially rated the RUF appearance (colour and texture), taste, smell, and mouth feeling, respectively, as good. Those who stated they disliked RUF taste perceived it as too salty (38%), too sour (24%), too greasy (24%) or too sweet (14%). Participants who disliked the mouth feeling stated it was too pasty (80%) or sticky (20%). However, up to 30% reported feeling at some point disgusted by RUF (Table 2), and 19% stated there had been occasions when they refused to eat it. At least one episode of diarrhoea and/or vomiting related to RUF intake was reported by 30% of participants at the first follow up visit, 2 weeks after initiating therapy. 24% of participants presented 1 to 3 episodes of diarrhoea and 12% presented 1 to 3 episodes of vomiting. The primary mode for eating RUF was directly from the sachet (85%) (Table 2). Many participants (63%) consumed RUF in place of breakfast, while others reported eating it just before (10%), or just after (12%) meals. Only 15% reported consuming RUF as a snack. At enrolment and week 2, 56 and 39% of participants, respectively, reached the minimum dietary diversity the day before the visit. One-third of the participants stated they hid from others when eating RUF (Table 2). The reasons given were fear of envious reactions (42%), arousing pity (19%), or teasing (16%), while 23% chose not to respond to the question. Occasional RUF sharing within the household was reported by 38% of caregivers and participants (Table 2). Caregivers reported that they felt: they (82%), other adults (53%), or other children (67%) in the household needed the RUF. Acceptability data were also compared by the severity of malnutrition and no remarkable association was found.

**Table 2:** Perceptions and behaviors related to the management of RUF therapy among undernourished HIV-infected participants and their caregiver 2 weeks after enrolment in the SNACS study, Senegal^a-b^

	< 12 years		≥ 12 years		All		*P* value
**Caregivers**							
Responsible for RUF management							<.0001
Caregiver	67	(81)	29	(33)	96	(56)	
Participant	16	(19)	60	(67)	76	(46)	
RUF sharing with caregiver	25	(30)	27	(30)	52	(30)	0.98
RUF sharing with other adults	6	(8)	6	(7)	12	(7)	0.83
RUF sharing with other children	13	(17)	11	(13)	24	(15)	0.42
**Participants**^**c**^							
RUF perceived as a drug							0.006
Yes	63	(76)	83	(93)	146	(85)	
No	8	(10)	3	(3)	11	(6)	
Don’t know	12	(14)	3	(3)	15	(9)	
Reason for RUF therapy							0.0005
Sickness	20	(24)	26	(30)	46	(27)	
Malnutrition	48	(58)	62	(71)	110	(64)	
Don’t know	15	(18)	–	–	15	(9)	
Participant needs encouragement for RUF feeding	39	(47)	33	(37)	72	(42)	0.19
Participant is disgusted by RUF	27	(33)	24	(27)	51	(30)	0.42
Participant hides to take RUF	21	(25)	29	(33)	50	(29)	0.29
Single sachet taken over several intake	44	(53)	36	(40)	80	(47)	0.10
Main mode of intake							0.18
Direct feeding from the sachet	67	(81)	79	(89)	146	(85)	
Diluted in gruel	14	(17)	10	(11)	24	(14)	
Spread on bread	2	(2)	–	–	2	(1)	

^a^Data are N (%)

^b^*RUF* ready-to-use food

^c^Questions were asked to the participant (and/or to the caregiver if need be when participant ≤7 years)

### Adherence to RUF

Most participants < 12 years were provided with 2 to 3 RUF sachets daily while most of their older counterparts received 3 to 4 sachets. If the maximum energy intake to be provided by RUF had not been limited to 2000 kcal/d, i.e. 4 sachets per protocol, 15 participants would have been prescribed 5 to 6 sachets according to weight and age prescription bands.

At week 2, 31% of participants were sub-optimal RUF consumers which means they had consumed less than 50% of their RUF provision. Only 11% of participants reported they had consumed all the doses prescribed.

Overall, participants reported having consumed 61% (45–81) of their RUF at week 2. This proportion was stable (varying between 64 and 57%) throughout the follow-up among the remaining participants. The energy provided by RUF per kg of body weight was significantly higher in MAM participants than in their SAM counterparts in both age group. However, energy intake from RUF per kg of body weight was far lower than expected based on the prescription weight bands for all participants; while being significantly higher in the younger participants than in their older counterparts (Table 3).

**Table 3:** Adherence to RUF among HIV-infected participants 2 weeks after enrolment in the SNACS study, Senegal ^a-b^

Indicators	< 12 years *N* = 84		≥ 12 years *N* = 89		*P*-Value
Sub-optimal RUF consumers, N (%)	24	(29)	30	(34)	0.46
% of RUF intake/provided	64	(46–90)	58	(42–74)	0.07
Energy provided/Kg of BW, Kcal/kg/d	61	(55–68)	55	(51–61)	< 0.0001
Energy intake/Kg of BW, Kcal/kg/d	39	(29–50)	31	(23–41)	0.001

^a^Data are median (IQR) unless otherwise indicated

^b^Abbreviations: *RUF* ready-to-use food, *BW* body weight

In both age groups, there was no difference either in proportions of sub-optimal RUF consumers or proportions of RUF intake and energy intake per kg of body weight according to the severity of malnutrition.

### Factors associated with sub-optimal RUF intake

To identify factors associated with early sub-optimal intake, we ran a stepwise logistic regression model with sub-optimal RUF intake (yes/no) as the dependent variable. Dislike of the taste of RUF (aOR = 5.0, 95% CI: 2.0–12.3), HIV-non disclosure (5.1, 1.9–13.9) and food insecurity (2.8, 1.1–7.2) were the major risk factors associated with early sub-optimal RUF intake (Table 4). There was no association between food insecurity and RUF taste appreciation, however, there was a significant interaction between these two variables in the multivariable model (*p* = 0.001). Stratified analysis indicated that in the absence of food insecurity, proportions of sub-optimal consumers were similar whatever the RUF taste appreciation (Mantel-Haenszel Chi2 test probability =0.36), while in food-insecure households, dislike of the taste of RUF was strongly associated with a sub-optimal intake (*p* < 0.0001).

**Table 4:** Risk factors associated with sub-optimal RUF intake in HIV-infected participants 2 weeks after enrolment in the SNACS Study.^a–b–c^, Senegal

Effects	Univariable analysis			Multivariable analysis		
	OR	95% CI	*P* value	aOR	95% CI	*P* value
Girls vs. boys	1.7	0.9–3.2	0.09	_	_	_
< 12 years vs ≥12 years	1.2	0.6–2.2	0.60	0.7	0.3–1.5	0.38
Decentralized setting vs Dakar	2.6	1.4–5.0	0.003	_	_	_
School level						
None vs. secondary	3.5	1.0–12.0	0.05	_	_	_
Primary vs. secondary	1.8	0.5–5.8	0.36	_	_	_
HIV status undisclosed: yes vs. no	4.9	2.1–11.2	0.0002	5.1	1.9–13.9	0.002
Food insecurity: yes vs. no	2.8	1.2–6.3	0.01	2.8	1.1–7.2	0.03
Disliking RUF taste: yes vs. no	3.6	1.7–7.8	0.001	5.0	2.0–12.3	< 0.001
Disliking RUF taste * Food insecurity	*_*	*_*	*_*	*_*	*_*	*0.001*
Caregiver responsible for RUF management vs. participant	2.2	1.1–4.1	0.02	_	_	_
Participant needs encouragement to eat the RUF: yes vs. no	2.0	1.0–3.6	0.04	_	_	_
Participant eats RUF in several vs. single feeding	1.8	1.0–3.3	0.06	_	_	_
SAM vs. MAM	1.9	1.0–3.5	0.05	_	_	_
Virologic suppression^d^: no vs. yes	2.4	1.2–4.6	0.01	2.0	0.9–4.4	0.07

^a^Abbreviations: *aOR* adjusted odds ratio, *CI* confidence interval, *OR* odds ratio, *RUF* ready-to-use food, *MAM* moderate acute malnutrition, *SAM* severe acute malnutrition

^b^Sub-optimal RUF intake is defined as if < 50% of RUF provided

^c^Explanatory variables are included at *P* < 0.20 in multivariate analysis, exited at *P* ≥ 0.10

^d^Virologic suppression is defined as viral load ≤50 copies/ml

### Expectations, perceptions and experiences of participants in the nutritional intervention

The qualitative analysis involved a convenience sub-sample of 24 participants in Dakar of whom 9 were girls and 8 were enrolled as SAM (Table 5). Four FGDs were conducted: two FGDs with participants who recovered within the study (one group with HIV-disclosed and one group with HIV-undisclosed participants), one in each Dakar study site and two FGDs with those who failed or defaulted using the same format. Discussions last 1 h30 on average. FGDs revealed that all the participants had similar expectations of the therapy. They were very concerned about their physical appearance, associating their thinness with being short and weak. The SAM participants reported they had been frequently left out of school sports programmes or street football games due to their poor fitness. Participants expressed their strong motivation to gain weight when they started the study. Those who recovered from wasting demonstrated satisfaction and pride in having gained weight. Since youths generally define themselves through the eyes of others, they also perceived the signs of their body shape’s normalization in friends’ comments: ‘*People tell me I’ve got big now! Big as a baobab tree! That I’ve got stronger and have calf muscles! That I’ve got more muscle!’* Boy, 9 years, HIV-undisclosed, recovery.

**Table 5:** Characteristics of HIV-infected participants included in the focus group discussions in the SNACS study, Senegal^a^

Characteristics	Participants *N* = 24
**Girl**	9
**Median age, year (min – max)**	13.7 (7.4–17)
**Acute malnutrition at enrollment**	
**Moderate**	16
**Severe**	8
**At school**	20
**Mother orphan**	13
**HIV disclosed**	15
**On ART**	20
**Outcome**	
**Recovery**	12
**Failure**	8
**Default**	4

^a^Data are N, unless otherwise indicated

Most participants reported that those around them were not told that they were participating in a nutrition intervention; a strategy recommended by health workers to avoid RUF sharing. Managing the daily intake of RUF out of sight proved complicated in homes - sometimes polygamous - where there is practically no space for young people; in particular for boys, to have some privacy: *‘I used to hide on the roof terrace to eat (RUF) because I’ve got a little brother who spends the day asking questions about everything he sees. If I don’t give him some he tells his mother and she tells me to give him some*.’ Boy, 12 years, HIV-disclosed, recovery*.* This daily effort by participants and caregivers to conceal consumption of RUF, however, is not always enough to avoid the sometimes necessary intra-familial sharing: ‘*My grandfather takes some from me, and sometimes he takes a lot. One day 12 sachets went missing.’* Boy, 16 years, HIV-undisclosed, failure, as well as extra-familial sharing, which is sometimes deliberate: *‘My friends used to ask me to give them some. I used to steal them from my grandmother’s bedroom and I hid them like that (he gestures) and I used to give some outside.’* Boy, 11 years, HIV-disclosed, recovery.

This secrecy, also motivated by the desire to divert friends’ curiosity and comments; even from well-meaning adults shows the secrecy that surrounds HIV infection and taking ARV: ‘*My friends used to ask me what I was taking. I didn’t tell them anything. You want to know my secret but I’m not going to say - because I can’t say!’* Boy, 14 years, HIV-disclosed, recovery. ‘Sometimes I meet people who tell me I’ve got bigger but I don’t tell them anything. I don’t want them to ask me.’ Boy, 8 years, HIV-undisclosed, failure.

Close follow-up visits at the hospital affected participants badly, worried that these repeated absences might arouse suspicion at school: *‘It used to bother me because I was absent too often. In the end, my classmates used to ask me what I had; sometimes it was my teachers. Only my teaching assistant knew that I was sick. It was my mother who went there. I don’t really know what she told them. My classmates used to ask me all the time why I was going to the hospital. I used to change the subject.’* Girl, 16 years, HIV-disclosed, recovery*.* Repeated absences could sometimes disrupt following classes and learning: *‘I lost marks because of my absences. Our lessons are long and catching up classes is complicated. It’s difficult to re-copy notes. It makes me tired.’* Boy, 12 years, HIV-disclosed, default.

The main divergence observed between participants who recovered and those who did not concerned the perception of RUF. The disgust of RUF, occurring early on or overtime, was a major constraint to RUF adherence: ‘*We have an illness [HIV-infection] and on top of that we have malnutrition. My friends are well-built; more than me. I feel that I’m malnourished and I am until now. At the start, I wanted it [RUF]. I forced myself; I used to take four (sachets) per day. I even liked it but over time it was the smell. It disgusted me.’* Boy, 16 years, HIV-disclosed, default.

Nausea, diarrhoea and vomiting whilst taking RUF were most often reported by unsuccessful participants for whom this represented a huge obstacle: ‘*The doctor asked me why I wasn’t putting on any weight. I told him every time I ate some I felt I was going to vomit. He said ‘Oh really?’ I replied yes. It was me that decided to give up.’* Girl, 8 years, HIV-undisclosed, failure.

Participants who recovered reported the various strategies they implemented to cope with the growing fatigue they felt in adhering to the RUF prescription, which consisted in alternating between modes of intake, or for some of the oldest, to allow themselves to have breaks of one to 2 days in RUF feeding. One participant, although affected by the study constraints, reorganized his daily routine around visits to the clinic and RUF feeding: ‘*I had to take three and I set the times: at 7:30 am I used to get up and take it. At 12:30 pm I used to leave the room to go into the bedroom. At 7:30 pm I used to go into the bedroom, heat the water, cut [the RUF] in the cup with water. I’d stir it and drink it. I think about my health and growth above all. I’ve got used to it.’* Boy, 13 years, HIV-disclosed, recovery.

## Discussion

Most participants initially reported a positive organoleptic appreciation of RUF. However, from the earliest weeks of the study, many of them needed encouragement from the caregiver for continued RUF feeding, and feelings of disgust were common. On average, participants reported having consumed two-third of their provision throughout the study, despite the adjustments made to reduce the recommended prescription. The rationale of the WHO prescription weight bands refers to an increase in the recommended nutritional intake levels for asymptomatic and malnourished HIV-infected people, which could reach 50 to 100% of energy needs in children [28]. The RUF is thus expected to provide a complete diet to a child with SAM with, in replacement of the habitual household diet, and cover additional energy needs due to HIV-infection [14]. Our results suggest that age- and weight-based prescription bands might have overestimated adolescents’ ability to consume RUF in “real life” and that there might be a threshold of RUF intake that some individuals cannot overcome due to nutritional density and sticky composition. Indeed, except for a few studies which proposed 2 sachets daily over short durations, in which HIV-adults stated that they appreciated RUF [19, 20] or adapted to the taste after the first weeks [29], a leading reason for non-adherence was disgust and growing tired of having to consume 3 to 4 sachets per day over a long time [16–18].

At week 2, 31% of participants reported having consumed less than half of the RUF provided. Not surprising, disliking the taste of the RUF was associated with sub-optimal RUF intake. This observation is consistent with studies involving HIV-infected adults reporting that many disliked the RUF taste and smell and that RUF taste and consistency made it difficult to swallow [17, 18, 29, 30]. Food insecurity is known as a major factor of poor adherence to food-by-prescription programmes as individuals are unlikely to refuse food ration sharing. Interestingly, stratified analyses showed that participants disliking RUF were more likely to present sub-optimal intake when they lived in food-insecure rather than in food-secure households. In case of participant reluctance, caregiver commitment to support RUF feeding might have been lower in the most vulnerable families, which thus encouraged sharing. The absence of association between sharing and sub-optimal intake could suggest that merely occasional sharing, perceived as acceptable, was reported. Group discussions with adolescents also suggest that some sharing escaped the attention of the caregivers.

We found HIV-non disclosure to be associated with sub-optimal RUF intake. This finding echoes our recent analysis in the same cohort reporting that HIV-disclosed participants showed a better understanding of the research information at enrolment in the present study [27]. Disease disclosure proceeds together with a better understanding of therapeutic issues, such as the importance of adherence to medication [31]. Such empowerment might have similarly benefited the early adherence to nutritional intervention.

The only other study which assessed similar support based on Plumpy Nut® among children and adolescents, conducted in an HIV clinic in Mali, reported greater adherence figures as 88% of participants were rated as “good” RUF consumers [32]. Comparisons between the two studies are however limited as they differ in prescription procedures. Also, the Malian study did not explore RUF sharing practice and acceptability of nutrition therapy and enrolled younger participants (< 15 years) with less severe acute malnutrition than we did.

The high prevalence of undesirable effects (nausea, vomiting and diarrhoea) attributed to RUF in this study is of concern as it hindered RUF acceptability and adherence in some participants. Qualitative studies also report similar undesirable effects attributed to RUF in adults on ART or starting ART and pregnant women [16, 17, 30]. In some of these patients, the initial undesirable effects were transient and attributed to pregnancy or drugs initiation. RUF feeding advice provided by the study staff, although partially followed, are questionable as they may not have been feasible in practice: for example to distribute RUF intake throughout the day and avoid mealtime. Many participants used to hide from others when eating RUF, most of the time in the caregiver’ bedroom, primarily to avoid being asked to share. Limited time to eat the RUF due to school and transportation and space constraints in the household combine to hinder the integration of feeding therapy into the usual food practices. In addition, many participants substituted their habitual breakfast with RUF or ate it at mealtime, with possible negative impacts on the dietary diversity provided by family meals. All in all, these constraints on RUF intake contribute to appetite saturation and decreased adherence.

This study has several limitations. First, the validity of information about both adherence and sharing practices is generally difficult to ensure, as some degree of socially desirable response bias might be expected. Counting the RUF sachets would have been a reliable method to confirm participants’ reports, but the pilot study showed little compliance with the instruction among participants and caregivers to return with unused sachets at each visit. Based on our mixed data analyses, we hypothesize that some participants’ reluctance to eat the RUF might have resulted in consistent family sharing, rather than regular sharing which would have reduced RUF feeding. Second, although RUF adherence data were computed at each follow-up point, we considered only the first 2 weeks of consumption to run the risk factors analyses. Early RUF adherence, while participants went through an adaptation phase, might not be representative of the overall RUF consumption. However, this methodological choice is justified for several reasons: (i) this avoided analytical bias due to cohort attrition (the first recoveries and defaults occurred at week 2), (ii) as in other studies [21, 32], adherence to RUF was rather stable over time among the remaining participants, (iii) adherence data coincide with acceptability data collected once at week 2. Third, we recruited participants in Dakar for the FGDs as these central sites have a longstanding and routine practice of hosting discussion groups as part of follow-up for older children and adolescents. By contrast, the regional sites had no comparable experience or practices. Although FGDs results may not be generalizable to all children and adolescents in Senegal, they highlight perceptions, behaviours, and constraints surrounding RUF use that are likely to be very similar in decentralized settings. Last, attendance at follow-up visits was demanding for our participants. However, because of our providing remuneration for travel costs, we documented very few missed or delayed visits. Attendance to follow-up visit and continuity of RUF provision might need specific attention in the routine care.

Palatability of imported RUF constitutes a major constraint for the acceptability of nutritional therapy. Studies comparing the acceptability of a novel and local product versus imported RUF (Plumpy Nut®) in HIV-infected or uninfected populations could not demonstrate the superiority of this alternative product so far [19, 33, 34]. Imported RUF is expected to remain by far the most widely used and easily available products for outpatient therapy. Therefore, an alternative lever for improving the acceptability of RUF therapy in routine care is to improve follow-up and support procedures.

The socially and physiologically acceptable RUF intake could be assessed with the young patient, and possibly adjusted during therapy according to their progression and tolerance. It would also facilitate increasing the time between follow-up visits to monthly or every other month since adolescents would not be given considerable RUF provision.

Compared to other age groups, HIV-infected adolescents exhibit higher rates of loss to follow up [35, 36], poor ART adherence [37, 38], and increased needs for psychosocial support [39]. Nutritional rehabilitation is thus an additional challenge to routine HIV care that requires adequate counselling and continuous support to ensure adherence, including preventing or lessening undesirable effects. Particular attention should be paid to adolescents with SAM - who were also older in this cohort- as they are likely to experience longer follow-up duration. This might lead to saturation and discouragement and strongly affect adherence to RUF-based protocols. Strengthening HIV clinic capacity and adolescent empowerment, through peer and community support and early HIV-disclosure process, could benefit nutritional interventions and improve the acceptability of RUF therapy.

Food-by-prescription is mostly used to focus on HIV-related wasting. Our study did not distribute food for the entire family to complement individual-targeted RUF therapy. Strategies to reduce sharing such as the rationalization of RUF prescription suggested above, the enrolment of other undernourished family members in RUF therapy, together with food complements for the household need to be evaluated.

## Conclusion

This study conducted at the national level in Senegal highlights several acceptability issues of RUF therapy based on WHO guidelines in HIV-infected children and adolescents, which may lead to decreasing adherence to therapy protocols. Beyond the initial barrier of RUF taste in some participants, the main factors affecting adherence were closely related to RUF feeding constraints and HIV non-disclosure. There is an urgent need for differentiated approaches to address specific health and nutritional needs of HIV-infected adolescents. Tailoring prescription guidance, empowering young patients in their care are and innovating in how to more effectively support them in their nutritional therapy are crucial levers for improving acceptability of RUF-based therapy in routine care. As such, our findings are valuable in informing improved management of nutritional care in this vulnerable population.

## Data Availability

The datasets generated and/or analyzed during the current study are available from the corresponding author on reasonable request.

## Abbreviations

ART: Antiretroviral treatment
BMIZ: Body mass index z-score (for age and sex)
FGD: Focus group discussion
IQR: Interquartile range
MAM: Moderate acute malnutrition
RUF: Ready-to-use food
SAM: Severe acute malnutrition

## References

[1] Gsponer T, Weigel R, Davies MA, Bolton C, Moultrie H, Vaz P (2012). Variability of growth in children starting antiretroviral treatment in southern Africa. Pediatrics.

[2] Weigel R, Phiri S, Chiputula F, Gumulira J, Brinkhof M, Gsponer T (2010). Growth response to antiretroviral treatment in HIV-infected children: a cohort study from Lilongwe, Malawi. Trop Med Int Health.

[3] WHO (2007). Community-based management of severe acute malnutrition. A Joint Statement by the World Health Organization, the World Food Programme, the United Nations System Standing Committee on Nutrition and the United Nations Children’s Fund.

[4] Fergusson P, Chinkhumba J, Grijalva-Eternod C, Banda T, Mkangama C, Tomkins A (2009). Nutritional recovery in HIV-infected and HIV-uninfected children with severe acute malnutrition. Arch Dis Child.

[5] Sadler K, Kerac M, Collins S, Khengere H, Nesbitt A (2008). Improving the management of severe acute malnutrition in an area of high HIV prevalence. J Trop Pediatr.

[6] Sunguya BF, Poudel KC, Mlunde LB, Otsuka K, Yasuoka J, Urassa DP (2012). Ready to use therapeutic foods (RUTF) improves undernutrition among ART-treated, HIV-positive children in Dar Es Salaam, Tanzania. Nutr J.

[7] Lowenthal ED, Bakeera-Kitaka S, Marukutira T, Chapman J, Goldrath K, Ferrand RA (2014). Perinatally acquired HIV infection in adolescents from sub-Saharan Africa: a review of emerging challenges. Lancet Infect Dis.

[8] Sohn AH, Hazra R (2017). Old problems for new providers: managing the Postpediatric HIV generation. Clin Infect Dis.

[9] Cames C, Pascal L, Diack A, Mbodj H, Ouattara B, Diagne NR (2017). Risk factors for growth retardation in HIV-infected Senegalese children on antiretroviral treatment: the ANRS 12279 MAGGSEN pediatric cohort study. Pediatr Infect Dis J.

[10] Jesson J, Masson D, Adonon A, Tran C, Habarugira C, Zio R (2015). Prevalence of malnutrition among HIV-infected children in central and west-African HIV-care programmes supported by the growing up Programme in 2011: a cross-sectional study. BMC Infect Dis.

[11] WHO (2014). Report of the consultation on the treatment of HIV among adolescents.

[12] WHO (2008). Essential prevention and care interventions for adults and adolescents living with HIV in resource-limited settings.

[13] Anema A, Zhang W, Wu Y, Elul B, Weiser SD, Hogg RS (2012). Availability of nutritional support services in HIV care and treatment sites in sub-Saharan African countries. Public Health Nutr.

[14] WHO (2009). Guidelines for an integrated approach to the nutritional care of HIV-infected children (6 months-14 years). Preliminary version for country introduction.

[15] Cavaleri MA, Kalogerogiannis K, McKay MM, Vitale L, Levi E, Jones S (2010). Barriers to HIV care: an exploration of the complexities that influence engagement in and utilization of treatment. Soc Work Health Care.

[16] Ali E, Zachariah R, Shams Z, Manzi M, Akter T, Alders P (2015). Peanut-based ready-to-use therapeutic food: how acceptable and tolerated is it among malnourished pregnant and lactating women in Bangladesh?. Matern Child Nutr.

[17] Dibari F, Bahwere P, Le Gall I, Guerrero S, Mwaniki D, Seal A (2011). A qualitative investigation of adherence to nutritional therapy in malnourished adult AIDS patients in Kenya. Public Health Nutr.

[18] Kebede MA, Haidar J (2014). Factors influencing adherence to the food by prescription program among adult HIV positive patients in Addis Ababa, Ethiopia: a facility-based, cross-sectional study. Infect Dis Poverty.

[19] Brown M, Nga TT, Hoang MA, Maalouf-Manasseh Z, Hammond W, Thuc TM (2015). Acceptability of two ready-to-use therapeutic foods by HIV-positive patients in Vietnam. Food Nutr Bull.

[20] Beckett AG, Humphries D, Jerome JG, Teng JE, Ulysse P, Ivers LC (2016). Acceptability and use of ready-to-use supplementary food compared to corn-soy blend as a targeted ration in an HIV program in rural Haiti: a qualitative study. AIDS Res Ther.

[21] Cames C, Varloteaux M, Have NN, Diom AB, Msellati P, Mbaye N (2016). Acceptability of outpatient ready-to-use food-based protocols in HIV-infected Senegalese children and adolescents within the MAGGSEN cohort study. Food Nutr Bull.

[22] WHO (2006). WHO child growth standards : length/height-for-age, weight-for-age, weight-for-length, weight-for-height and body mass index-for-age : methods and development.

[23] WHO (2013). Guideline: Updates on the management of severe acute malnutrition in infants and children.

[24] Nutriset. Nutritional Solutions' References Malaunay, France: Nutriset; [cited 2015 1s2/10/2015]. Available from: http://www.nutriset.fr/en/product-range/scientific-litterature/nutritional-solutions-references.html.

[25] Coates J, Swindale A, Bilinsky P (2007). Household food insecurity access scale (HFIAS) for measurement of household food access: Indicator guide (v. 3).

[26] FAO (2014). Introducing the minimum dietary diversity – women (MDD-W) global dietary diversity Indicator for women.

[27] Hejoaka F, Varloteaux M, Desclaux-Sall C, Ndiaye SM, Diop K, Diack A (2019). Improving the informed consent process among HIV-infected undisclosed minors participating in a biomedical research: insights from the multicentre nutritional SNACS study in Senegal. Tropical Med Int Health.

[28] WHO (2003). Nutrient requirements for people living with HIV/AIDS: report of a technical consultation.

[29] Olsen MF, Tesfaye M, Kaestel P, Friis H, Holm L (2013). Use, perceptions, and acceptability of a ready-to-use supplementary food among adult HIV patients initiating antiretroviral treatment: a qualitative study in Ethiopia. Patient Prefer Adherence.

[30] Hussein S, Worku A, Aklilu A, Abate K (2015). Ready-to-use therapeutic food for Management of Wasting in HIV infected adults: a qualitative investigation of views and experiences of patients in Ethiopia. Int J Nutr Food Sci.

[31] Nichols J, Steinmetz A, Paintsil E (2017). Impact of HIV-status disclosure on adherence to antiretroviral therapy among HIV-infected children in resource-limited settings: a systematic review. AIDS Behav.

[32] Jesson J, Coulibaly A, Sylla M, N'Diaye C, Dicko F, Masson D (2017). Evaluation of a nutritional support intervention in malnourished HIV-infected children in Bamako, Mali. J Acquir Immune Defic Syndr.

[33] Dibari F, Bahwere P, Huerga H, Irena AH, Owino V, Collins S (2013). Development of a cross-over randomized trial method to determine the acceptability and safety of novel ready-to-use therapeutic foods. Nutrition.

[34] Owino VO, Irena AH, Dibari F, Collins S (2014). Development and acceptability of a novel milk-free soybean-maize-sorghum ready-to-use therapeutic food (SMS-RUTF) based on industrial extrusion cooking process. Matern Child Nutr.

[35] Auld AF, Agolory SG, Shiraishi RW, Wabwire-Mangen F, Kwesigabo G, Mulenga M (2014). Antiretroviral therapy enrollment characteristics and outcomes among HIV-infected adolescents and young adults compared with older adults--seven African countries, 2004-2013. MMWR Morb Mortal Wkly Rep.

[36] Lamb MR, Fayorsey R, Nuwagaba-Biribonwoha H, Viola V, Mutabazi V, Alwar T (2014). High attrition before and after ART initiation among youth (15-24 years of age) enrolled in HIV care. AIDS.

[37] Fokam J, Sosso SM, Yagai B, Billong SC, Djubgang Mbadie RE, Kamgaing Simo R (2019). Viral suppression in adults, adolescents and children receiving antiretroviral therapy in Cameroon: adolescents at high risk of virological failure in the era of "test and treat". AIDS Res Ther.

[38] Nachega JB, Hislop M, Nguyen H, Dowdy DW, Chaisson RE, Regensberg L (2009). Antiretroviral therapy adherence, virologic and immunologic outcomes in adolescents compared with adults in southern Africa. J Acquir Immune Defic Syndr.

[39] Denison JA, Banda H, Dennis AC, Packer C, Nyambe N, Stalter RM (2015). "the sky is the limit": adhering to antiretroviral therapy and HIV self-management from the perspectives of adolescents living with HIV and their adult caregivers. J Int AIDS Soc.

